# Comparative evaluation of early treatment with ceftolozane/tazobactam versus ceftazidime/avibactam for non-COVID-19 patients with pneumonia due to multidrug-resistant *Pseudomonas aeruginosa*

**DOI:** 10.1093/jac/dkae313

**Published:** 2024-09-11

**Authors:** Thomas P Lodise, Engels N Obi, Alexandre H Watanabe, Emre Yucel, Jae Min, Brian H Nathanson

**Affiliations:** Department of Pharmacy Practice, Albany College of Pharmacy and Health Sciences, 106 New Scotland Avenue, Albany, NY, USA; Merck & Co., Inc., 2025 E Scott Ave, Rahway, NJ, USA; Merck & Co., Inc., 2025 E Scott Ave, Rahway, NJ, USA; Merck & Co., Inc., 2025 E Scott Ave, Rahway, NJ, USA; Merck & Co., Inc., 2025 E Scott Ave, Rahway, NJ, USA; OptiStatim LLC, 25 Willow Circle, Longmeadow, MA, USA

## Abstract

**Background:**

Ceftolozane/tazobactam and ceftazidime/avibactam are commonly used in patients with MDR-*Pseudomonas aeruginosa* (PSA) pneumonia (PNA). This study compared outcomes between non-COVID-19 hospitalized patients with MDR-PSA PNA who received ceftolozane/tazobactam or ceftazidime/avibactam.

**Methods:**

The study included non-COVID-19 adult hospitalized patients with MDR-PSA PNA in the PINC AI Healthcare Database (2016–22) who received ceftolozane/tazobactam or ceftazidime/avibactam within 3 days of index culture for ≥2 days. Outcomes were mortality, recurrent MDR-PSA PNA, discharge destination, post-index culture day length of stay (LOS) and costs (in US dollars, USD), and hospital readmission.

**Results:**

The final sample included 197 patients (117 ceftolozane/tazobactam, 80 ceftazidime/avibactam). No significant differences were observed in mortality and post-index culture LOS and costs between groups. In the multivariable analyses, patients who received ceftolozane/tazobactam versus ceftazidime/avibactam had lower recurrent MDR-PSA PNA (7.9% versus 18.0%, *P* = 0.03) and 60 day PNA-related readmissions (11.1% versus 28.5%, *P* = 0.03) and were more likely to be discharged home (25.8% versus 9.8%, *P* = 0.03). Compared with ceftazidime/avibactam patients, ceftolozane/tazobactam patients had lower adjusted median total antibiotic costs (5052 USD versus 8099 USD, *P* = 0.003) and lower adjusted median comparator (ceftolozane/tazobactam or ceftazidime/avibactam) antibiotic costs (3938 USD versus 6441 USD, *P* = 0.005). In the desirability of outcome ranking (DOOR) analysis, a ceftolozane/tazobactam-treated patient was more likely to have a more favourable outcome than a ceftazidime/avibactam-treated patient [DOOR probability: 59.6% (95% CI: 52.5%–66.8%)].

**Conclusions:**

Early treatment with ceftolozane/tazobactam may offer some clinical and cost benefits over ceftazidime/avibactam in patients with MDR-PSA PNA. Further large-scale studies are necessary to comprehensively understand the outcomes associated with these treatments for MDR-PSA PNA.

## Introduction

Hospital-acquired bacterial pneumonia (HABP) and ventilator-associated bacterial pneumonia (VABP) account for 22% of all HAIs^1^ and are associated with substantial morbidity and mortality.^2^*Pseudomonas aeruginosa* is one of the most frequent causative pathogens of HABP/VABP^3^ and a conventional antipseudomonal β-lactam (e.g. cefepime, piperacillin/tazobactam or meropenem) is routinely used for the empirical treatment of patients with suspected or documented HABP/VABP due to *P. aeruginosa*.^4–6^ The efficacy of conventional antipseudomonal β-lactams for treating patients with *P. aeruginosa* HABP/VABP has been limited by the emergence of MDR *P. aeruginosa* (MDR-PSA) isolates.^7–9^ Two currently preferred agents for treating MDR-PSA pneumonia (PNA) in adults are ceftolozane/tazobactam and ceftazidime/avibactam.^10,11^ Both agents are approved for the treatment of adult patients with HABP/VABP caused by *P. aeruginosa* and other Gram-negative pathogens.^12–15^ These agents exhibit high *in vitro* microbiological activity against MDR-PSA in surveillance studies^16–18^ and both have been associated with improved effectiveness and/or safety compared with aminoglycoside- or polymyxin-based combinations in real-world evidence studies of patients with highly resistant *P. aeruginosa* infections.^19–23^ However, there are limited published clinical comparative studies between ceftolozane/tazobactam and ceftazidime/avibactam in patients with MDR-PSA PNA.^24,25^ The primary objective of this study was to compare clinical and healthcare resource utilization (HRU) outcomes between non-COVID-19 adult hospitalized patients with MDR-PSA PNA patients who received early treatment^26–28^ either ceftolozane/tazobactam or ceftazidime/avibactam across various US hospitals.

## Methods

### Study design and population

A retrospective, multicentre observational study of adult hospitalized patients in the PINC AI™ Healthcare Data (PHD),^29^ from January 2016 to February 2020 and from January 2021 to September 2022, was performed. Due to poor COVID-19 documentation between March and December 2020, adult hospitalized patients in PHD were excluded from the study during this period. PHD, formerly known as the Premier Healthcare Database, is a large, US hospital-based, service-level, all-payer database that contains information on inpatient discharges, primarily from geographically diverse non-profit, non-governmental and community and teaching hospitals and health systems from rural and urban areas (Table S1, available as Supplementary data at *JAC* Online).^29^

Patients from the PHD during the specified time periods were included in this study if they met the following criteria: (i) age ≥ 18 years; (ii) evidence of clinical diagnosis for PNA based on International Classification of Diseases, 10th revision (ICD-10) diagnostic codes;^30^ (iii) identification of MDR-PSA (first isolate with resistant or intermediate antibiotic susceptibility to at least one agent in three classes of antipseudomonal antibiotics)^8^ on a clinical blood or respiratory culture consistent with PNA; (iv) receipt of any IV antibiotic(s) 2 days prior to index MDR-PSA culture collection day (−2 days) to ≤3 days after index MDR-PSA collection day (+3 days); (v) receipt of ceftolozane/tazobactam or ceftazidime/avibactam within 3 days post-index MDR-PSA PNA culture collection day; and (vi) treatment with ceftolozane/tazobactam or ceftazidime/avibactam for >2 days. We required patients to receive ≥2 days of ceftolozane/tazobactam or ceftazidime/avibactam within 3 days of index MDR-PSA PNA culture collection day as previous studies have demonstrated that a minimum of 2 days of treatment is required for an assessment of treatment-related outcomes.^26–28^

Patients were excluded from the study if they had any of the following: (i) diagnosis of cystic fibrosis or moderate to severe bronchiectasis; (ii) hospital length of stay (LOS) of <2 days post-index MDR-PSA culture collection day; (iii) transferred from another acute care facility with MDR-PSA on a clinical culture; (iv) missing in-hospital mortality or hospital cost data; (v) simultaneous receipt of ceftolozane/tazobactam and ceftazidime/avibactam in the 3 days post-index MDR-PSA PNA culture collection day; and (vi) documented positive SARS-CoV-2 test or COVID-19 diagnosis. Finally, only the first encounter was considered among patients with ≥1 MDR-PSA PNA episode that met the study criteria during the study period. This study utilized already existing Health Insurance Portability and Accountability Act (HIPAA)-compliant fully de-identified data and was exempt from Institutional Review Board (IRB) review.^29^

### Baseline data covariates and outcomes

Hospital-level variables included US census region, hospital bed size, teaching status (teaching versus non-teaching) and population served (urban versus rural). Patient-level variables included information on demographics, medical history, hospitalization course, microbiology, infection type and medications received. Description of baseline covariates are shown in Table S2.

Clinical outcomes assessed included recurrent MDR-PSA PNA during the index hospitalization, 30 day mortality post-index culture collection day, in-hospital mortality, discharge destination (home versus other), and 30 day and 60 day hospital readmissions (all-cause and PNA/sepsis-related). PNA/sepsis-related readmission was determined based on ICD diagnosis or diagnosis-related group (DRG) codes for PNA and sepsis (Table S3). HRU outcomes evaluated were hospital LOS from index MDR-PSA PNA culture collection day to hospital discharge and inflation-adjusted hospital costs from index MDR-PSA PNA culture collection day to hospital discharge. Fixed and variable costs assigned by PHD were used to determine hospital costs from index MDR-PSA PNA culture collection day to hospital discharge for each patient (Table S4). For the purposes of the analyses, hospital costs from index MDR-PSA PNA culture collection day to hospital discharge were computed overall to reflect the total cost to treat the patient from index MDR-PSA PNA culture collection day to hospital discharge. Hospital costs from index MDR-PSA PNA culture collection day to hospital discharge were also computed for the following subcategories: room and board, pharmacy, antibiotics (a subset of the pharmacy costs), ceftolozane/tazobactam or ceftazidime/avibactam costs, and all other medical care. Room and board included the fixed costs associated with the hospital room in which the patient resided each day. Pharmacy costs reflected the costs of all pharmacotherapies received by the patient. Other medical care reflected the costs associated with all other healthcare services and procedures other than pharmacotherapies and room and board. All costs before 2022 were adjusted to reflect 2022 US dollars (USD) using values from the Federal Reserve inflation calculator.^31^

Given the importance of understanding the association between benefits and harms when determining the utility of a treatment, a ‘within-patient’ or composite outcome analysis using the desirability of outcome ranking (DOOR) strategy was performed.^32–36^ Consistent with published research,^35^ each study patient was assigned a mutually exclusive rank of 1 to 5 (Table S5). Rank 1 represented the most desirable outcome and included anyone who was discharged home alive and did not experience any of the undesirable, pre-specified events. Rank 5 represented the least desirable outcome and included all patients who died during their hospitalization. Ranks 2 to 4 include patients who were discharged alive but had 1, 2 or 3 events, respectively. The events included in the DOOR analysis were as follows: hospital survivor but not discharged home; recurrent MDR-PSA PNA; and 30 day PNA/sepsis-related readmission.

### Statistical methods

Bivariate analyses were first performed to compare baseline characteristics between treatment groups and outcomes between treatment groups. As part of the bivariate treatment-outcomes analyses, subgroup analyses were performed among (i) patients with difficult-to-treat (DTR)-PSA PNA;^37^ (ii) patients receiving mechanical ventilation (MV) on index MDR-PSA PNA culture collection day; and (iii) patients in the ICU on index MDR-PSA PNA culture collection day. For the overall and subgroup analyses, Student’s *t*-test was used to compare means between two groups and the Kruskal–Wallis test was used to compare continuous distributions non-parametrically between two groups. The chi-squared test was used to compare frequencies by groups unless a cell count (expected number of patients) was <5, wherein the Fisher exact test was used. Kaplan–Meier survival curves were used to compare the time to in-hospital mortality between treatment groups, and survivor functions of the treatment groups were compared with the log-rank test.

Multivariate analyses were performed to determine the association between treatment and each outcome while adjusting for potential confounding variables. Least absolute shrinkage and selection operator (LASSO) regression modelling was used for variable selection among a set of plausible confounders determined *a priori* (Table S6). Treatment with ceftolozane/tazobactam or ceftazidime/avibactam were forced in each model and the other covariates included in each final model was determined by the LASSO algorithm to prevent overfitting. Logistic regression was used to examine binary outcomes. Generalized linear models with a logarithmic link and a gamma distribution were used to examine continuous outcomes due to the skewed distributions of these outcomes.

The DOOR distribution was compared between treatment groups in the overall population and pre-specified subgroups of interest, and the probability of a more desirable outcome with one treatment compared to the other (DOOR probability; Wilcoxon Mann–Whitney *U* statistic adjusted for tie) was calculated with a corresponding two-sided 95% CI.^38^ Like previous studies,^33,35^ a partial credit analysis using three different scenarios [score of 1 (100%) to the most desirable category of the ordinal outcome, 0 (0%) to the least desirable, and partial credit to the intermediate categories] was performed (Table S2). All analyses were done using Stata/MP 18.0 for Windows (StataCorp LLC, College Station, TX, USA). *P* values of <0.05 were considered statistically significant.

## Results

During the study period, 197 patients met the study criteria (Table S7). Among the 197 patients, 117 (59.4%) received ceftolozane/tazobactam and 80 (40.6%) received ceftazidime/avibactam. The median (IQR) treatment duration was 9 (6–15) days in the ceftolozane/tazobactam group and 7 (4–10) days in the ceftazidime/avibactam group. In the ceftolozane/tazobactam group, 31 (26.5%) patients received concomitant therapy (therapy post-index on the same service day) with an aminoglycoside for ≥2 days and 14 (12.0%) patients received concomitant therapy with a polymyxin for ≥2 days. In the ceftazidime/avibactam group, 19 (23.8%) patients received concomitant therapy with an aminoglycoside for ≥2 days and 9 (11.3%) patients received concomitant therapy with a polymyxin for ≥2 days. Antibiotic susceptibility results were available in 73 (62.4%) and 18 (22.5%) of the ceftolozane/tazobactam- and ceftazidime/avibactam-treated patients, respectively. Among the 73 ceftolozane/tazobactam-treated patients with ceftolozane/tazobactam susceptibility results, 62 (85%) were susceptible. Among the 18 ceftazidime/avibactam-treated patients with ceftazidime/avibactam susceptibility results, 11 (61%) were susceptible. The index MDR-PSA PNA culture was susceptible to either meropenem, cefepime or tazobactam/piperacillin in 13 out of the 44 ceftolozane/tazobactam-treated patients with missing ceftolozane/tazobactam susceptibility results and 27 out of the 62 ceftazidime/avibactam-treated patients with missing ceftazidime/avibactam susceptibility results.

Table 1 presents comparisons of baseline characteristics between treatment groups. Overall, treatment groups were similar at baseline with few exceptions. Unadjusted and adjusted treatment-outcome comparisons for the overall study population are shown in Table 2. Variables included in each LASSO regression model are provided in Table S6. In the multivariable analyses, no significant differences in in-hospital mortality and 30 day mortality were observed between treatment groups. Similarly, no difference in time to in-hospital mortality was observed between groups in the Kaplan–Meier analyses (Figure S1). Patients who received ceftolozane/tazobactam versus ceftazidime/avibactam were significantly more likely to be discharged home and were less likely to have a recurrent MDR-PSA PNA. No differences in adjusted median post-index MDR-PSA culture collection day hospital LOS and total hospital costs and incidences of all-cause readmissions and 30 day PNA/sepsis-related readmissions were observed between the two treatment groups. Compared with ceftazidime/avibactam patients, ceftolozane/tazobactam patients had significantly lower adjusted median (IQR) post-index MDR-PSA culture collection day total antibiotic costs and lower median (IQR) post-index MDR-PSA culture collection day comparator (ceftolozane/tazobactam or ceftazidime/avibactam) antibiotic costs. Ceftolozane/tazobactam-treated patients were also found to have significantly lower 60 day PNA/sepsis-related readmissions relative to ceftazidime/avibactam-treated patients. The adjusted treatment-outcome comparisons for the pre-specified subgroups of interest aligned with the results in the overall study population with few exceptions (Table S8).

**Table 1.** Comparison of baseline characteristics between ceftolozane/tazobactam and ceftazidime/avibactam in the overall study population

Characteristic	Ceftolozane/tazobactam	%	Ceftazidime/avibactam	%	*P* value
	*n* = 117		*n* = 80		
US census region					
Midwest	37	31.6	22	27.5	
Northeast	6	5.1	6	7.5	0.357
South	70	59.8	45	56.3	
West	4	3.4	7	8.8	
Hospital bed size					
<300	26	22.2	23	28.8	
300–499	24	20.5	29	36.3	0.006
500+	67	57.3	28	35.0	
Teaching hospital	77	65.8	41	51.3	0.041
Urban location of hospital	106	90.6	69	86.3	0.341
Age (years), mean (SD)	60.9 (13.8)		60.4 (12.9)		0.759
Sex: male	82	70.1	50	62.5	0.266
Race					
White	82	70.1	52	65.0	
Black	24	20.5	21	26.3	0.663
Other	7	6.0	3	3.8	
Unknown	4	3.4	4	5.0	
Admission source					
Non-healthcare facility (including home)	76	65.0	49	61.3	
Clinic	8	6.8	5	6.3	0.902
Transfer from SNF, ICF	17	14.5	16	20.0	
Transfer from a different hospital	12	10.3	8	10.0	
Transfer from another non-acute care facility or other/unknown	4	3.4	2	2.5	
Primary payer					
Medicare	65	55.6	48	60.0	
Medicaid	25	21.4	22	27.5	
Managed care	21	17.9	8	10.0	0.435
Commercial/worker’s comp/self-pay	4	3.4	1	1.3	
Other	2	1.7	1	1.3	
Hospitalization in the 6 months prior to the index MDR-PSA PNA admission	54	46.2	46	54.5	0.118
Charlson comorbidity index					
Mean (SD)	3.4 (2.3)		3.9 (3.4)		0.157
Median (IQR)	3 (1–5)		4 (2–5)		0.188
Charlson comorbidities					
Acute myocardial infarction	17	14.5	11	13.8	0.878
Congestive heart failure	49	41.9	29	36.3	0.427
Peripheral vascular disease	13	11.1	8	10.0	0.804
Cerebrovascular disease	20	17.1	16	20.0	0.604
Dementia	8	6.8	8	10.0	0.425
COPD	49	41.9	34	42.5	0.931
Rheumatoid disease	7	6.0	3	3.8	0.743
Peptic ulcer disease	3	2.6	4	5.0	0.445
Mild liver disease	5	4.3	3	3.8	1.000
Diabetes	24	20.5	14	17.5	0.599
Diabetes with complications	24	20.5	25	31.3	0.087
Hemiplegia or paraplegia	17	14.5	19	23.8	0.100
Renal disease	40	34.2	36	45.0	0.126
Cancer	8	6.8	4	5.0	0.765
Moderate/severe liver disease	3	2.6	1	1.3	0.648
Metastatic cancer	2	1.7	2	2.5	1.000
AIDS	1	0.9	0	0.0	1.000
Hospital LOS prior to index MDR-PSA culture day					
Mean (SD)	9.0 (13.0)		9.5 (17.9)		0.849
Median (IQR)	4 (1–11)		2 (1–8)		0.157
Residence in ICU on index MDR-PSA PNA culture day	73	62.4	56	70.0	0.270
Receipt of MV on index MDR-PSA culture day	67	57.3	57	71.3	0.046
Presence of PSA on a clinical culture prior to index MDR-PSA culture collection day	29	24.8	11	13.8	0.059
Presence of bloodstream infection with a non-MDR-PSA pathogen within 30 days of index MDR-PSA PNA culture day	10	8.5	15	18.8	0.035
Presence of concurrent MDR-PSA bloodstream infection ±3 days of index MDR-PSA culture day	12	10.3	5	6.3	0.325
Index MDR-PSA culture met DTR criteria	41	35.0	22	27.5	0.265
Index MDR-PSA culture was carbapenem resistant	112	95.7	69	86.3	0.017
Index MDR-PSA Culture was polymicrobial (excluding *Staphylococcus* spp./*Streptococcus* spp.)	26	22.2	29	36.3	0.031
Index MDR-PSA culture was polymicrobial (including *Staphylococcus* spp./*Streptococcus* spp.)	33	28.2	34	42.5	0.038
Infection type					
nvHABP	38	32.5	14	17.5	
vHABP	45	38.5	48	60.0	0.009
VABP	34	29.1	18	22.5	
Antibiotics received between admission and index MDR-PSA culture day					
Aminoglycoside^a^	25	21.4	14	17.5	0.503
β-Lactam (older)^b^	104	88.9	65	81.3	0.132
Fluoroquinolone	19	16.2	11	13.8	0.633
Newer β-lactam/β-lactam-β-lactamase inhibitor^c^	43	36.8	43	53.8	0.018
Polymyxin	5	4.3	4	5.0	1.000
Vancomycin	93	79.5	60	75.0	0.458
Daptomycin	5	4.3	3	3.8	1.000
Other glycopeptide/glycopeptide-like agent^d^	1	0.9	0	0.0	1.000
Macrolide^e^	12	10.3	5	6.3	0.325
Oxazolidinone	13	11.1	20	25.0	0.010
Rifamycin	0	0.0	0	0.0	1.000
Sulpha-like agent^f^	9	7.7	3	3.8	0.366
Tetracycline-like agent^g^	14	12.0	7	8.8	0.473
Number of antibiotics received between admission and index MDR-PSA culture day					
0	2	1.7	0	0.0	
1	7	6.0	7	8.8	
2	42	35.9	21	26.3	0.272
3	32	27.4	31	38.8	
≥4	34	29.1	21	26.3	
Other antibiotics received from index C/T or CZA treatment day until hospital discharge					
Aminoglycoside	43	36.8	30	37.5	0.915
β-Lactam (older)^b^	79	67.5	61	76.3	0.185
Fluoroquinolone	22	18.8	11	13.8	0.351
Other newer β-lactam or β-lactam/β-lactam-β-lactamase inhibitor^c^	16	13.7	11	13.8	0.988
Polymyxin	18	15.4	20	25.0	0.093
Vancomycin	70	59.8	58	72.5	0.067
Daptomycin	12	10.3	4	5.0	0.288
Other glycopeptide/glycopeptide-like agent^d^	0	0.0	0	0.0	1.000
Macrolide^e^	7	6.0	5	6.3	0.939
Oxazolidinone	26	22.2	15	18.8	0.555
Rifamycin	0	0.0	0	0.0	1.000
Sulpha-like agent^f^	11	9.4	4	5.0	0.288
Tetracycline-like agent^g^	18	15.4	12	15.0	0.941

SNF, skilled nursing facility; ICF, intermediate care facility; nvHABP, non-ventilator-associated HABP; vHABP, ventilator-associated HABP; C/T, ceftolozane/tazobactam; CZA, ceftazidime/avibactam.

^a^Aminoglycosides included gentamicin, tobramycin and amikacin.

^b^Older β-lactams included amoxicillin/clavulanate, amoxicillin, ampicillin, ampicillin/sulbactam, aztreonam, bacampicillin, carbenicillin, cefaclor, cefadroxil, cefamandole, cefazolin, cefdinir, cefditoren pivoxil, cefepime, cefixime, cefonicid, cefoperazone, cefotaxime, cefotetan, cefoxitin, cefpodoxime, cefprozil, ceftaroline, ceftibuten, ceftizoxime, ceftriaxone, cefalexin, cefapirin, dicloxacillin, doripenem, ertapenem, imipenem, loracarbef, meropenem, mezlocillin, penicillin, piperacillin, piperacillin/tazobactam, ticarcillin/clavulanate and ticarcillin.

^c^Newer β-lactam/β-lactam-β-lactamase inhibitors included ceftolozane/tazobactam, ceftazidime/avibactam, imipenem/relebactam, cefiderocol and meropenem/vaborbactam.

^d^Other glycopeptide/glycopeptide-like agents included dalbavancin, oritavancin and telavancin.

^e^Macrolides included azithromycin, clarithromycin, erythromycin/sulfisoxazole and quinupristin/dalfopristin.

^f^Sulpha-like drugs included sulfamethoxazole, sulfamethoxazole/trimethoprim, trimethoprim and sulfisoxazole.

^g^Tetracycline-like drugs included doxycycline, eravacycline, minocycline, omadacycline and tigecycline.

**Table 2.** Comparison of unadjusted and adjusted outcomes between ceftolozane/tazobactam and ceftazidime/avibactam in overall study population

Outcomes	C/T *n* = 117	C/A *n* = 80	Unadjusted *P* value	Adjusted C/T *n* = 117	Adjusted C/A *n* = 80	Adjusted *P* value
In-hospital mortality, % (95% CI)	17.9 (11.5–26.1)	22.5 (13.9–33.2)	0.431	15.9 (9.5–22.2)	26.8 (15.2–38.4)	0.168
30 Day mortality, % (95% CI)	13.7 (8.0–21.3)	16.3 (8.9–26.2)	0.616	14.0 (8.2–19.8)	15.7 (8.5–22.9)	0.731
Discharged home versus other, % (95% CI)	24.8 (17.3–33.6)	7.5 (2.8–15.6)	0.002	25.8 (18.5–33.0)	9.8 (2.8–16.8)	0.009
Recurrent MDR-PSA PNA, % (95% CI)	7.7 (3.6–14.1)	18.8 (10.9–29.0)	0.020	7.9 (3.1–12.6)	18.0 (10.3–25.7)	0.030
Post-index MDR-PSA PNA culture collection day LOS, days, median (95% CI)	14.0 (11.0–16.0)	10.0 (8.0–14.3)	0.219	13.7 (10.3–17.1)	12.7 (8.6–16.8)	0.723
Post-index MDR-PSA PNA culture collection day total costs, USD, median (95% CI)	$45 607 (37 062–54 964)	$53 978 (38 182–61 800)	0.787	$62 857 (53 561–72 154)	$54 440 (43 126–65 754)	0.269
Post-index MDR-PSA PNA culture collection day room & board costs, USD, median (95% CI)	$21 164 (18 213–25 003)	$23 654 (18 695–29 070)	0.791	$28 877 (22 422–35 333)	$25 788 (17 928–33 649)	0.559
Post-index MDR-PSA PNA culture collection day total pharmacy costs, USD, median (95% CI)	$9640 (8320–13 501)	$13 486 (11 525–16 182)	0.196	$12 240 (9808–14 671)	$14 611 (11 638–17 584)	0.241
Post-index MDR-PSA PNA culture collection day antibiotic costs, USD, median (95% CI)	$5082 (4391–6008)	$8062 (6890–9898)	0.023	$5052 (3828–6275)	$8099 (6612–9587)	0.003
Post-index MDR-PSA PNA culture collection day C/T or C/A costs, USD, median (95% CI)	$3497 (3049–4344)	$6337 (3766–7202)	0.032	$3938 (2862–5014)	$6441 (5133–7749)	0.005
Post-index MDR-PSA PNA culture collection day other costs, USD, median (95% CI)	$12 072 (10 696–14 591)	$12 230 (8530–16 203)	0.720	$15 194 (11 996–18 392)	$14 304 (10 413–18 194)	0.733

Readmission outcomes for survivors	*n* = 96	*n* = 62		*n* = 96	*n* = 62	
30 Day all-cause readmission, % (95% CI)	21.9 (14.1–31.5)	19.4 (10.4–31.4)	0.704	17.3 (10.8–23.9)	29.2 (17.1–41.3)	0.119
60 Day all-cause readmission, % (95% CI)	27.1 (18.5–37.1)	27.4 (16.9–40.2)	0.963	23.0 (15.2–30.8)	35.7 (22.5–48.8)	0.113
30 Day pneumonia/sepsis-related readmission, % (95% CI)	9.4 (4.4–17.1)	9.7 (3.6–19.9)	0.950	8.3 (3.3–13.4)	15.8 (4.9–26.7)	0.223
60 Day pneumonia/sepsis-related readmission, % (95% CI)	13.5 (7.4–22.0)	16.1 (8.0–27.7)	0.653	11.1 (5.5–16.6)	28.5 (15.4–41.7)	0.031

C/T, ceftolozane/tazobactam; CZA, ceftazidime/avibactam.

**Figure 1.**
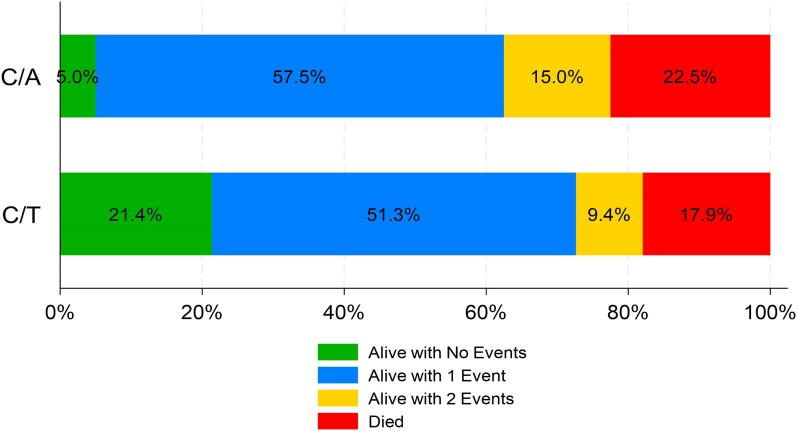
Comparison of desirability of outcome ranking distribution between ceftolozane/tazobactam and ceftazidime/avibactam in the overall study population. Rank 1 represented the most desirable outcome and included anyone who was discharged home alive and did not experience any of the undesirable, pre-specified events. Rank 5 represented the least desirable outcome and included all patients who died during their hospitalization. Ranks 2 to 4 include patients who were discharged alive but had 1, 2 or 3 events, respectively. The events included in the DOOR analysis were as follows: hospital survivors who were not discharged home; recurrent MDR-PSA PNA; and 30 day PNA/sepsis-related readmission.

The distribution of DOOR rankings in the overall study population by treatment is shown in Figure 1. More ceftolozane/tazobactam-treated patients were discharged home with no events relative to ceftazidime/avibactam-treated patients (21.4% versus 5%, respectively, *P* = 0.002). Results of the DOOR analyses are shown in Table 3. In the overall population, the probability that a patient in the ceftolozane/tazobactam group would have a more favourable outcome than a patient in the ceftazidime/avibactam group was 59.6% (95% CI: 52.5%–66.8%). In the pre-specified subgroup DOOR analyses, a patient in the ceftolozane/tazobactam group also had a higher probability of a more favourable outcome than a patient in the ceftolozane/tazobactam group. However, the entire 95% CI associated with each DOOR probability in the subgroup analyses only exceeded 50% (DOOR probability of 50% indicated no difference) among patients with DTR-PSA PNA. Results of the DOOR partial credit analyses in overall study population are shown in Table 4. In scenario A (represents a patient who values only hospital survival), the mean partial credit scores were not significant difference between treatment groups. In contrast, the mean partial credit score was significantly higher in the ceftolozane/tazobactam group versus the ceftazidime/avibactam group in scenario B (represents a patient who places more value on minimizing events and would not accept any undesirable event). In scenario C (represents a patient who places significant value on survival but balances this with wanting to avoid some events), the mean partial credit score was higher in the ceftolozane/tazobactam group versus the ceftazidime/avibactam group, but the difference in mean scores was not significant between treatment groups.

**Table 3.** Comparison of DOOR probabilities between ceftolozane/tazobactam and ceftazidime/avibactam in the overall study population and pre-specified subgroups of interest

Group	*n*	DOOR probability (%)^a^	95% CI
Overall cohort	197	59.6	(52.5–66.8)
Patients with DTR-PSA PNA	63	61.9	(50.1–73.6)
Patients who were on MV on index MDR-PSA culture collection day	145	58.1	(49.8–66.5)
Patients who resided in an ICU on index MDR-PSA culture collection day	129	54.9	(45.9–64.0)

^a^DOOR probability of >50% indicates a more desirable outcome in ceftolozane/tazobactam-treated patients relative to ceftazidime/avibactam-treated patients. DOOR probability of <50% indicates a more desirable outcome in ceftazidime/avibactam-treated patients relative to ceftolozane/tazobactam-treated patients. DOOR probability of 50% indicates no difference in outcome between treatment groups.

**Table 4.** DOOR partial credit analysis in overall study population

Scenario	Mean C/T score	Mean CZA score	Difference in DOOR scores (C/T−CZA)	95% CI	Welch *t*-test *P* value
A	82.1	77.5	4.6	−7.1–16.2	0.441
B	21.4	5.0	16.4	7.4–25.3	<0.001
C	64.5	55.6	8.9	−0.4–18.2	0.061

Scenario A represents a patient who values only hospital survival (equivalent to a mortality outcome). Scenario B represents a patient who places more value on minimizing events and would not accept any undesirable event. Scenario C represents a patient who places significant value on survival but balances this with wanting to avoid some events. For each scenario, the mean of the partial credits scores is calculated for each treatment group and then the difference between groups is obtained. A difference with a 95% CI that overlaps zero indicates no significant difference between groups.

C/T, ceftolozane/tazobactam; CZA, ceftazidime/avibactam.

## Discussion

There were several notable findings in this retrospective, multicentre, comparative effectiveness study of non-COVID-19 adult hospitalized patients with MDR-PSA PNA who received early treatment^26–28^ with either ceftolozane/tazobactam or ceftazidime/avibactam. Not surprisingly, no difference in mortality was observed between treatment groups. While all-cause mortality is the primary endpoint for approval of agents for treatment of patients with HABP/VABP,^39^ death is an insensitive endpoint for discriminating therapies for patients with HABP/VABP given the heterogeneity and high underlying risk of death in this population.^40–44^ Mortality in patients with MDR-PSA HABP/VABP is influenced by many factors unrelated to the targeted therapeutic intervention and lack of differences in mortality between therapies does not exclude the existence of clear and sufficient evidence of benefit or harm.^40–44^ In support of this notion, a recent systematic review found scant evidence of any pharmacological intervention conferring a consistent mortality benefit in critically ill patients.^45^ An additional consideration in this study when evaluating mortality as an endpoint is that all patients were required to receive early therapy with either ceftolozane/tazobactam or ceftazidime/avibactam. We purposefully limited the study to those who received ceftolozane/tazobactam or ceftazidime/avibactam within 3 days of index MDR-PSA culture collection to maximize internal validity and minimize any biases that may have been introduced by prolonged receipt of other concomitant antipseudomonal agents^39^ or by failure to receive treatment with ceftolozane/tazobactam or ceftazidime/avibactam in a timely manner.^26–28^ This further minimized our ability to detect any differences between treatments for non-specific, multicausal outcome measures like mortality.^46^

Although no difference in mortality was observed between treatments, patients who received ceftolozane/tazobactam versus ceftazidime/avibactam were less likely to have recurrent MDR-PSA PNA (adjusted difference of 11%) and a 60 day PNA/sepsis-related readmission (adjusted difference of 15%). From an adjusted number-needed-to-treat perspective (Table S9), the findings suggest that 1 out of 10 MDR-PSA PNA and 1 out of 6 60 day PNA/sepsis-related readmissions could have potentially been avoided if ceftolozane/tazobactam versus ceftazidime/avibactam was prescribed. Patients who received ceftolozane/tazobactam versus ceftazidime/avibactam-treated patients were also more likely to be discharged home (25.8% versus 9.8%, adjusted difference of 16%, *P* = 0.009). In most cases, hospital survivors who were not discharged home required additional care in a long-term care facility (Table S10). Due to the nature of PHD, we were unable to determine the reason(s) that led to the patient’s discharge destination. However, based on an adjusted difference of 16%, this finding suggests that approximately 1 in 6 ceftazidime/avibactam-treated patients may have had worse functional status at hospital discharge relative to those who received ceftolozane/tazobactam given their need for continued care in a healthcare facility post-hospitalization.^47,48^ Additionally, lower adjusted median antibiotic and comparator post-index culture collection were observed in ceftolozane/tazobactam- versus ceftazidime/avibactam-treated patients. However, no significant differences in post-index culture collection LOS and total costs were observed between the two groups. Like mortality, many non-pharmacological factors contribute these outcomes in patients with HABP/VABP^46^ and these likely explained, in part, the observed null post-index culture collection LOS and total costs findings.

The potential global outcomes associated with ceftolozane/tazobactam relative to ceftazidime/avibactam in this study were best highlighted in the DOOR analysis,^32–36^ which suggested that there was a 59.6% (95% CI: 52.5%–66.8%) probability a patient in the ceftolozane/tazobactam group would have a more favourable outcome than a patient in the ceftazidime/avibactam group. Among patients with DTR-PSA PNA, one of the primary infection types for which ceftolozane/tazobactam or ceftazidime/avibactam is recommended for first-line use,^10,11^ the probability of a more favourable outcome with ceftolozane/tazobactam relative to ceftazidime/avibactam was 61.9% (95% CI: 50.1%–73.6%). From a clinical standpoint, the findings from the DOOR analyses were best captured in the partial credit analyses, which suggested that ceftolozane/tazobactam-treated patients were more likely to be discharged home with no events relative to ceftazidime/avibactam-treated patients. Although extreme caution should be exercised when interpreting the results given the retrospective nature of the data, the collective findings suggest that early treatment with ceftolozane/tazobactam may offer some potential clinical and cost benefits over ceftazidime/avibactam in patients with MDR-PSA PNA. However, further large-scale studies are necessary to comprehensively understand the outcomes associated with these treatments for MDR-PSA PNA.

Several issues should be noted when interpreting the study findings. Treatments were not randomly assigned and there is a potential for biases due to differences in patients’ severity and unmeasured cofounders between treatment group. Study design restrictions, stratified analyses and multivariable regression modelling were used to minimize the influence of the potential systematic biases. However, these methods cannot account for unmeasured confounders and a reasonable degree of caution should be exercised when interpreting the results. Due to the nature of PHD, we also had to purposefully limit study outcomes to include only objective measures like PNA/sepsis-related readmissions versus MDR-PSA-related readmissions to minimize any subjective biases that may result from assessing and interpreting electronic healthcare data.^49^

Observed ceftolozane/tazobactam or ceftazidime/avibactam susceptibility data were limited in PHD. Since there was a low prevalence of carbapenemase-producing *P. aeruginosa* in the USA during the study period, we anticipate that most MDR-PSA had simultaneous expression of up-regulated efflux pump(s), down-regulated or deleted porin(s) and/or hyperproduction of class C AmpC β-lactamases versus acquired serine-β-lactamases or MBLs.^50^ Unfortunately, the hospitals within PHD are de-identified^29^ and we are unable to ascertain any additional information on ceftolozane/tazobactam or ceftazidime/avibactam susceptibility across institutions. We were also unable to examine more clinically relevant outcomes like emergence of resistance to ceftolozane/tazobactam or ceftazidime/avibactam during therapy given the limited *P. aeruginosa* susceptibility data on newer β-lactam agents in PHD. Emergence of resistance on treatment is a particularly relevant outcome given the increased reports of such cases among patients with MDR-PSA infections and should be an endpoint in future comparative studies.^51–53^ Additionally, we were unable to assess the potential impact of dosing regimens received on the observed treatment-outcomes analyses as only limited data on antibiotic dosing were available in the PHD. Lastly, a third of the study population had polymicrobial PNA. While this finding is consistent with some other recent comparative effectiveness studies,^19–25^ the presence of polymicrobial PNA may have limited the generalizability of the findings. As such, there is a need for future, large-scale comparative effectiveness studies between ceftolozane/tazobactam and ceftazidime/avibactam among patients with MDR-PSA PNA with complete ceftolozane/tazobactam and ceftazidime/avibactam dosing and susceptibility data and bacterial genomic data. This will be particularly relevant for ceftolozane/tazobactam as its approved dosing varies by indication.^15^

The algorithm that was used to identify MDR-PSA HABP/VABP cases in the PHD was designed to optimize the specificity of the MDR-PA HABP/VABP diagnoses. The potential for misclassification exists, particularly as we did not have access to chest X-ray data to corroborate the HABP/VABP diagnosis.^54–56^ There is also the potential that some positive MDR-PSA cultures may have represent colonization rather than true PNA. Of note, such misclassification would likely have diluted the effects of treatment on the observed outcomes and thus we believe the potential for misclassification has a minimal effect on the observed findings. Additionally, we only had culture and antibiotic susceptibility results per the local hospital laboratory report in PHD. We were unfortunately unable to determine which MIC testing methodology was used or which susceptibility breakpoints were employed across sites. We anticipate VITEK or MicroScan were used to determine susceptibility results at most hospitals and the FDA breakpoint recommendations were followed, given the use of an automatic susceptibility device, but we are not certain.

Lastly, this study was limited to non-COVID-19 adult hospitalized patients with MDR-PSA PNA who received ceftolozane/tazobactam or ceftazidime/avibactam within 3 days of index culture for ≥2 days to minimize the any potential confounding/biases associated with COVID-19^57^ and/or delayed receipt of ceftolozane/tazobactam or ceftazidime/avibactam therapy^26–28^. It is unclear if the results are applicable to COVID-19 patients or those who receive treatment with ceftolozane/tazobactam or ceftazidime/avibactam >3 days after index MDR-PSA culture collection day. As stated above, a third of the study population had polymicrobial PNA and this may somewhat limit the generalizability of the finding. These aspects of our study design may have also accounted for the discordant findings between this and other studies that compared the outcomes between ceftolozane/tazobactam and ceftazidime/avibactam for the treatment of patients with *P. aeruginosa* infections.^24,25^

In conclusion, the collective findings from this study suggest that early treatment with ceftolozane/tazobactam may offer some potential clinical and cost benefits over ceftazidime/avibactam in non-COVID-19 adult, hospitalized patients with MDR-PSA PNA. There is a critical need for studies of this nature to help inform clinical decisions for patients with MDR-PSA HABP/VABP given the limitations associated with the registrational HABP/VABP randomized controlled trials (RCTs). Caution should be exercised when interpreting the results given the retrospective nature of the data and further large-scale studies are necessary to comprehensively understand the outcomes associated with these treatments for patients with MDR-PSA PNA.

## Supplementary Material

dkae313_Supplementary_Data

## Transparency declarations

TPL: AbbVie (Consultant), Cidara (Advisor/Consultant), Ferring (Advisor/Consultant/Speaker), Genentech (Consultant), GlaxoSmithKline (Consultant), ICPD (Consultant), Melinta (Advisor/Consultant), Merck (Advisor/Consultant, Grant/Research Support), Paratek (Advisor/Consultant), Roche (Consultant), Seres (Consultant), Shionogi (Advisor/Consultant/Speaker), Wockhardt (Grant/Research Support), and Venatorx (Advisor/Consultant). BHN: Merck (Consultant), EviMed (Consultant). ENO, AHW, EY and JM are employees of Merck Sharp & Dohme LLC, a subsidiary of Merck & Co., Inc., Rahway, NJ, USA.

## Supplementary data

Figure S1 and Tables S1 to S10 are available as Supplementary data at *JAC* Online.
