# Supplementary material for: Comparative evaluation of early treatment with ceftolozane/tazobactam versus ceftazidime/avibactam for non-COVID-19 patients with pneumonia due to multidrug-resistant Pseudomonas aeruginosa
Source: J Antimicrob Chemother. 2024 Sep 11;79(11):2954–64. doi: 10.1093/jac/dkae313 (PMC11531822; doi:10.1093/jac/dkae313)
Supplement: dkae313_Supplementary_Data [file dkae313_supplementary_data.docx]

**Supplemental Figure 1.** Comparison of Time to In-Hospital Mortality Between Ceftolozane/Tazobactam and Ceftazidime/Avibactam in Overall Study Population

**Supplemental Table 1.** Description of PINC AI™ Healthcare Data (PHD)

“PINC AI™ Healthcare Data (PHD), formerly known as the Premier Healthcare Database, is a large, U.S. hospital-based, service-level, all-payer database that contains information on inpatient discharges, primarily from geographically diverse non-profit, nongovernmental and community and teaching hospitals and health systems from rural and urban areas (**Table S1**). Hospitals/healthcare systems submit administrative, healthcare utilization and financial data from patient encounters. It includes over 121 million inpatient visits with more than 10 million per year since 2012, representing approximately twenty-five percent of annual United States inpatient admissions. Relevant to this study, the PINC AI Healthcare Database contains information on hospital and visit characteristics; patient demographics and disease states; admission and discharge diagnoses; pharmacy data, microbiologic and laboratory tests performed, diagnostic and therapeutic services, overall and departmental hospital costs (fixed and variable), and patient disposition and discharge health status.^1^”

**Supplemental Table 2.** Description of Baseline Covariates

Patient demographics included age, sex, race, admission source, and primary payer. Medical history included hospitalization ≤ 6 months of index admission and Charlson Comorbidity Index (CCI) (overall score and individual conditions).^2^ Data collected during hospital course included hospital length of stay (LOS) prior to index MDR-PSA PNA culture collection day, residence in an ICU on index MDR-PSA PNA culture day, and receipt of mechanical ventilation (MV) on index MDR-PSA PNA culture day. Microbiologic data included presence of PSA on a clinical culture prior to MDR-PSA PNA index date during index admission, presence of a bloodstream infection(s) for a non-MDR-PSA pathogen within 30 days of index MDR-PSA PNA culture day during index MDR-PSA PNA admission, culture and antibiotic susceptibility results for index MDR-PSA culture per local hospital laboratory report, presence of difficult-to-treat (DTR-PSA)^3^ or carbapenem resistance on index MDR-PSA PNA culture per local hospital laboratory report, presence of a concurrent MDR-PSA bloodstream infections ± 3 days of index MDR-PSA PNA culture, and presence of other non-MDR-PSA pathogens on index MDR-PSA PNA culture (polymicrobial PNA). Pneumonia infection types included non-ventilated HABP (nvHABP), ventilated HABP (vHABP), and VABP.^4^ Antibiotic(s) received between admission and index MDR-PSA culture collection day, duration of ceftolozane/tazobactam or ceftazidime/avibactam treatment, and other antibiotics received from index ceftolozane/tazobactam or ceftazidime/avibactam treatment allocation day through discharge were documented.

**Supplemental Table 3.** ICD diagnosis or DRG codes for Pneumonia and Sepsis

Pneumonia or Sepsis related re-admission for survivors is defined as a readmission with the following MS DRG or ICD-10 Code as a primary diagnosis

**DRG Description**

177 RESPIRATORY INFECTIONS ND INFLAMMATIONS WITH MCC

178 RESPIRATORY INFECTIONS AND INFLAMMATIONS WITH CC

179 RESPIRATORY INFECTIONS AND INFLAMMATIONS WITHOUT CC/MCC

853 INFECTIOUS AND PARASITIC DISEASES WITH O.R. PROCEDURE WITH MCC

854 INFECTIOUS AND PARASITIC DISEASES WITH O.R. PROCEDURE WITH CC

855 INFECTIOUS AND PARASITIC DISEASES WITH O.R. PROCEDURE WITHOUT CC/MCC

856 POSTOPERATIVE OR POST-TRAUMATIC INFECTIONS WITH O.R. PROCEDURE WITH MCC

857 POSTOPERATIVE OR POST-TRAUMATIC INFECTIONS WITH O.R. PROCEDURE WITH CC

858 POSTOPERATIVE OR POST-TRAUMATIC INFECTIONS WITH O.R. PROCEDURE WITHOUT CC/MCC

862 POSTOPERATIVE AND POST-TRAUMATIC INFECTIONS WITH MCC

863 POSTOPERATIVE AND POST-TRAUMATIC INFECTIONS WITHOUT MCC

870 SEPTICEMIA OR SEVERE SEPSIS WITH MV >96 HOURS

871 SEPTICEMIA OR SEVERE SEPSIS WITHOUT MV >96 HOURS WITH MCC

872 SEPTICEMIA OR SEVERE SEPSIS WITHOUT MV >96 HOURS WITHOUT MCC

| **ICD-10 code** | **Description** |
| --- | --- |
| A48.1 | Legionnaires' disease |
| A22.1 | Pulmonary anthrax |
| A37.91 | Whooping cough, unspecified species with pneumonia |
| B25.0 | Cytomegaloviral pneumonitis |
| B44.0 | Invasive pulmonary aspergillosis |
| J13 | Streptococcus pneumoniae |
| J14 | Pneumonia due to Hemophilus influenzae |
| J15 | Bacterial pneumonia, not elsewhere classified |
| J15.8 | Pneumonia due to other specified bacteria |
| J15.9 | Unspecified bacterial pneumonia |
| J16 | Pneumonia due to other infectious organisms, not elsewhere classified |
| J16.8 | Pneumonia due to other specified infectious organisms |
| J17 | Pneumonia in diseases classified elsewhere |
| J18 | Pneumonia, unspecified organism |
| J18.0 | Bronchopneumonia, unspecified organism |
| J18.1 | Lobar pneumonia, unspecified organism |
| J18.2 | Hypostatic pneumonia, unspecified organism |
| J18.8 | Other pneumonia, unspecified organism |
| J18.9 | Pneumonia, unspecified organism |
| J22 | Unspecified acute lower respiratory infection |
| J44.0 | Chronic obstructive pulmonary disease with (acute) lower respiratory infection |
| J10.0 | Influenza due to other identified influenza virus with pneumonia |
| J10.00 | Influenza due to other identified influenza virus with unspecified type of pneumonia |
| J10.08 | Influenza due to other identified influenza virus with other specified pneumonia |
| J11.00 | Influenza due to unidentified influenza virus with unspecified type of pneumonia |
| J11.08 | Influenza due to unidentified influenza virus with specified pneumonia |
| J15.1 | Pneumonia due to Pseudomonas |
| J15.5 | Pneumonia due to *Escherichia coli* |
| J15.6 | Pneumonia due to other aerobic Gram-negative bacteria |
| J95.851 | Ventilator-associated pneumonia |
| R65.20 | Severe sepsis without septic shock |
| R65.21 | Severe sepsis with septic shock |

**Supplemental Table 4.** Description of Hospital Cost Calculations in PINC AI™ Healthcare Data (PHD)

Fixed and variable costs assigned by PHD were used to determine hospital costs from index MDR-PSA PNA culture collection day to hospital discharge for each patient. Fixed costs included those that did not relate directly to or vary with the activity (volume) of the department such as depreciation, management, repair and maintenance and overhead. Variable costs included expenses that related directly to or vary with the activity (volume) of the department such as supplies and “hands-on” patient care activities. Data on all items (i.e., hospital services, medical procedures, equipment fees, supplies, drugs, and diagnostic evaluations such as imaging and laboratory tests) billed to a patient from index MDR-PSA PNA culture collection day to hospital discharge were used to determine variable costs. In PHD, each billable item has an assigned unique identifier code, and an associated cost is provided in PHD to determine the costs associated with each variable cost item.

**Supplemental Table 5.** Generalized DOOR Analysis Strategy

| **Rank** | **Alive at hospital discharge** | **How many of the following events:**  **1. Discharge to a higher acuity location**  **2. Recurrent MDR-PSA PNA**  **3. 30-day PNA-related readmission** |
| --- | --- | --- |
| 1 (most desirable) | Yes, and discharged to the same/similar admission point of origin | 0 of 3 |
| 2 | Yes | 1 of 3 |
| 3 | Yes | 2 of 3 |
| 4 | Yes | 3 of 3 |
| 5 (least desirable) | No (death) | Any |

Rank 1 represented the most desirable outcome and included anyone who was discharged alive to home and did not experience any of the undesirable, pre-specified events. Rank 5 represented the least desirable outcome and included all patients who died during their hospitalization. Ranks 2 through 4 include patients who were discharged alive but had 1, 2, or 3 events, respectively. The events included in the DOOR analysis were as follows: discharged alive to home, recurrent MDR-PSA PNA, and 30-day PNA/sepsis-related readmission.

**DOOR Partial Credit Scoring in Scenarios A, B, and C**

| Door Rank | Scenario A (full credit) | Scenario B (no credit) | Scenario C (partial credit) |
| --- | --- | --- | --- |
| Alive and no events | 100 | 100 | 100 |
| Alive with 1 event | 100 | 0 | 75 |
| Alive with 2 events | 100 | 0 | 50 |
| Alive with 3 events | 100 | 0 | 25 |
| Death | 0 | 0 | 0 |

Scenario A represents a patient who values only hospital survival (equivalent to a mortality outcome). Scenario B represents a patient who places more value on minimizing events and would not accept any undesirable event. Scenario C represents a patient who places significant value on survival but balances this with wanting to avoid some events. For each scenario, the mean of the partial credits scores is calculated for each treatment group and then the difference between groups is obtained. A difference with a 95% CI that overlaps zero indicates no significant difference between groups.

Abbreviations: CI, confidence interval; DOOR, desirability of outcome ranking C/T, ceftolozane/tazobactam; CZA, ceftazidime/avibactam.

**Supplemental Table 6:** Variables included at Model Entry in the Least absolute shrinkage and selection operator (LASSO) regression models

|  | **In-hospital Morality** | **30 Day Mortality** | **MDR-PSA Recurrence** | **Discharged Home (New Definition)** | **30-Day All-Cause Readmission** | **60-Day All-Cause Readmission** | **30-Day PNA-Related Readmission** | **60-Day PNA-Related Readmission** | **Post-Culture Collection Day LOS** | **Post- Culture Collection Total Costs** | **Post- Culture Collection Total Pharmacy Cost** | **Post-Culture Collection Room & Board Costs** | **Post-Culture Collection Other Costs** | **Post-Culture Collection Antibiotic Costs** | **Post-Culture Collection C/A or C/T Cost** |
| --- | --- | --- | --- | --- | --- | --- | --- | --- | --- | --- | --- | --- | --- | --- | --- |
| **Treatment (C/T vs. C/A)** | **X** | **x** | **X** | **X** | **X** | **X** | **X** | **X** | **X** | **X** | **X** | **X** | **X** | **X** | **X** |
| **US Census Region** | x |  |  | x |  | x | x |  |  |  | x | x |  |  |  |
| **Hospital Bed Size** | x |  |  |  | x |  | x |  |  |  |  |  |  |  |  |
| **Teaching Status** | x |  |  |  | x |  |  | x |  |  |  |  |  |  |  |
| **Age** | x | x | x | x |  |  |  |  | x | x | x | x |  |  |  |
| **Sex** |  |  |  |  | x | x | x | x |  |  |  |  |  |  |  |
| **Race** | x | x |  | x |  |  |  |  | x |  |  | x |  |  |  |
| **Admission Source** | x |  |  | x | x |  |  |  |  |  |  |  |  |  |  |
| **Charlson Comorbidity Index** | x | x |  |  |  |  | x | x |  |  |  |  |  |  |  |
| **Hospital LOS from Admission to Index MDR-PSA Culture Day** | x |  | x |  | x | x | x | x | x | x | x | x | x |  |  |
| **Residence in ICU on Index MDR-PSA Culture Day** | x | x |  | x |  |  | x | x |  |  |  | x |  |  |  |
| **Receipt of MV on Index MDR-PSA Culture Day** |  |  |  | x | x | x | x | x |  |  |  |  |  |  |  |
| **Infection Type** | x |  | x | x | x |  | x | x | x | x | x | x |  |  |  |
| **Presence of a Concurrent MDR-PSA Bloodstream Infections ± 3 Days of Index MDR-PSA Culture Day** | x |  |  | x |  |  | x | x | x | x |  | x | x |  |  |
| **Index MDR-PSA Culture met DTR Criteria** |  |  |  |  |  |  |  |  |  | x | x |  |  |  |  |
| **Index MDR-PSA was carbapenem resistance** | x |  |  |  |  |  |  |  |  |  |  |  |  |  |  |
| **Aminoglycosides on or after Index Culture** | x |  |  |  |  |  |  |  | x |  | x |  |  |  |  |
| **Fluoroquinolones on or after Index Culture** | x |  | x | x |  |  |  |  | x |  |  |  |  |  |  |
| **Glycopeptide/glycopeptide-like agents on or after Index Culture** | x |  |  | x | x | x | x | x |  |  |  |  |  |  |  |
| **Beta Lactam (excluding C/T and C/A) on or after Index Culture** |  |  |  |  |  |  |  | x |  |  |  |  |  |  |  |
| **All other Antibiotics on or After Index Culture** |  |  |  |  |  |  |  |  |  |  |  |  |  |  |  |
| **Pre-Covid vs. Post-Covid period** | x |  |  |  | x | x |  | x |  |  |  |  |  |  |  |

**Supplemental Table 7.** Study Attrition Table

| **Inclusion criteria** | **N** | **%** |
| --- | --- | --- |
| Documented inpatient hospitalization with discharge date between Jan 2016- Feb 2020 (pre-COVID-19 period) and Jan 2021-Sep 2022 (post COVID-19 period). Note period between Mar-Dec 2020 was not included due to poor COVID-19 documentation & Age ≥ 18 years old and hospitals are submitting laboratory values. | 1,996,780 | 100% |
| Evidence of clinical diagnosis of pneumonia (PNA) by ICD-10 codes | 304,720 | 15.3% |
| Patient has *Pseudomonas aeruginosa* based on laboratory results | 33,287 | 1.7% |
| Patient has a valid respiratory or blood culture source | 24,177 | 1.2% |
| Patient has multiple drug resistance in their respiratory or blood culture P. *aeruginosa* | 5,012 | 0.3% |
| Patient had a valid Index Culture specimen day (e.g., culture was drawn as an inpatient) | 4,960 | 0.2% |
| Receipt of any IV antibiotic(s) 2 days prior to index MDR-PSA culture collection day (-2 days) to ≤ 3 days after index MDR-PSA collection day (+3 days). | 4,150 | 0.2% |
| Receipt of ceftolozane/tazobactam or ceftazidime/avibactam within 5 days post-index MDR-PSA PNA culture collection day and treatment with ceftolozane/tazobactam or ceftazidime/avibactam was continued for >2 days. | 648 | 0.0% |
| **Exclusion Criteria** |  | % of 648 |
| Diagnosis of cystic fibrosis or moderate to severe bronchiectasis per ICD-10 codes | 593 | 91.5% |
| Patients with missing in-hospital mortality or hospital cost data. | 589 | 90.9% |
| Hospital LOS <2 days post index MDR-PSA culture collection day | 588 | 90.7% |
| Patient transferred from another acute care facility with an index MDR-PSA culture within -1 to +3 days of hospital admission | 551 | 85.0% |
| Patients with a documented inpatient hospitalization with discharge date between 2021-2022 and a documented positive SARS-CoV-2 test and or COVID-19 discharge diagnosis. COVID-19 patients were excluded to minimize the potential of selection bias and confounding in outcome analyses. | 551 | 85.0% |
| Patients who received both ceftolozane/tazobactam and ceftazidime/avibactam within 5 days and ceftolozane/tazobactam and ceftazidime/avibactam were received for same number of days during the 5-day post-index MDR-PSA PNA culture collection day window. | 526 | 81.2% |
| Patients with multiple valid encounters, only the first encounter is analyzed. | 492 | 75.9% |
| Patients with ceftolozane/tazobactam or ceftazidime/avibactam first provided more than 2 service days from index culture | 197 | 30.4% |

**Supplemental Table 8.** Comparison of Unadjusted and Adjusted Outcomes Between Ceftolozane/Tazobactam and Ceftazidime/Avibactam Across Pre-Specified Subgroups of Interest

|  | **Residence in ICU on Index MDR-PSA Culture Day** | | | **Receipt of MV on Index MDR-PSA Culture Day** | | | **DTR-PSA PNA** | | |
| --- | --- | --- | --- | --- | --- | --- | --- | --- | --- |
| **Outcomes** | **C/T**  **N =73** | **CZA**  **N =53** | **P-value** | **C/T**  **N = 79** | **CZA**  **N = 66** | **P-value** | **C/T**  **N = 41** | **CZA**  **N = 22** | **P-value** |
| **In-Hospital Mortality** | 26.0% | 26.8% | 0.923 | 24.1% | 25.8% | 0.813 | 12.2% | 18.2% | 0.707 |
| **30-Day Mortality** | 19.2% | 19.6% | 0.947 | 17.7% | 18.2% | 0.943 | 12.2% | 13.6% | 1.000 |
| **Discharged Home vs. Other** | 17.8% | 3.6% | 0.013 | 19.0% | 4.5% | 0.011 | 24.4% | 0.0% | 0.011 |
| **Recurrent MDR-PSA PNA** | 9.6% | 16.1% | 0.268 | 10.1% | 22.7% | 0.043 | 4.9% | 13.6% | 0.333 |
| **Mean (95% CI) Post-Index Length of Stay** | 15.6 (11.4, 19.8) | 21.7 (14.9, 28.6) | 0.160 | 19.3 (13.9, 24.7) | 21.7 (15.4, 28.1) | 0.565 | 12.5 (7.1, 17.8) | 14.1 (10.7, 17.6) | 0.575 |
| **Median [IQR] Post Index Total Costs, in USD** | 54442 [33095, 113402] | 53145 [29867, 90813] | 0.342 | 61766 [33435, 113056] | 58113 [32687, 91414] | 0.623 | 40645 [26434, 67661] | 37826 [22715, 66684] | 0.634 |
| **Median [IQR] Post Index Total Room & Board Costs, in USD** | 23780 [15319, 55213] | 24503 [10743, 44467] | 0.364 | 26791 [15426, 48973] | 24503 [11900, 44937] | 0.654 | 18902 [11824, 33162] | 20363 [8461, 31911] | 0.445 |
| **Median [IQR] Post Index Total Pharmacy Costs, in USD** | 13369 [6486, 25860] | 13322 [6727, 20632] | 0.690 | 13385 [6724, 25476] | 13749 [8442, 21746] | 0.940 | 8375 [5670, 16932] | 13127 [6785, 16046] | 0.507 |
| **Median [IQR] Post Index Antibiotic Costs, in USD** | 5232 [3056, 11531] | 7757 [2809, 11450] | 0.635 | 5274 [2869, 11531] | 8503 [4267, 11669] | 0.182 | 4473 [2575, 9722] | 8422 [3083,11828] | 0.116 |
| **Median [IQR] Post Index C/T or CZA Costs, in USD** | 3711 [1819, 8544] | 4201 [1785, 8753] | 0.669 | 3979 [1819, 8114] | 6439 [2302, 9905] | 0.146 | 3219 [1550, 6027] | 5299 [3083, 8264] | 0.166 |
| **Median [IQR] Post Index Total Other Costs, in USD** | 14628 [9244, 34578] | 11823 [7498, 28077] | 0.121 | 14871 [9244, 34142] | 15544 [8247, 29602] | 0.418 | 10945[7178, 21088] | 8433 [5309, 15930] | 0.210 |
| **30-Day All Cause Readmission among Survivors** | 22.2% | 19.5% | 0.748 | 15.0% | 16.3% | 0.849 | 19.4% | 22.2% | 0.811 |
| **60-Day All Cause Readmission among Survivors** | 25.9% | 29.3% | 0.717 | 20.0% | 26.5% | 0.420 | 30.6% | 27.8% | 0.833 |
| **30-Day Pneumonia/Sepsis-Related Readmission among Survivors** | 9.3% | 7.3% | 1.000 | 5.0% | 8.2% | 0.698 | 5.6% | 11.1% | 0.594 |
| **60-Day Pneumonia/Sepsis-Related Readmission among Survivors** | 11.1% | 14.6% | 0.609 | 8.3% | 16.3% | 0.200 | 13.9% | 16.7% | 1.000 |

**Supplemental Table 9:** Adjusted Number Needed to Treat with Ceftolozane/Tazobactam and Ceftazidime/Avibactam to Avoid 1 Outcome

| **Clinical Outcomes** | **Adjusted Number Needed to Treat** |
| --- | --- |
| **In-Hospital Mortality** | 9.1; NS |
| **30-Day Mortality** | 61.0; NS |
| **Discharged Home vs. Other** | **6.3; 95% CI (3.8, 17.5)** |
| **Recurrent MDR-PSA PNA** | **9.9; 95% CI (5.2, 100.4)** |
| **30-Day All Cause Readmission** | 8.4; NS |
| **60-Day All Cause Readmission** | 7.9; NS |
| **30-Day Pneumonia/Sepsis-Related Readmission** | 13.5; NS |
| **60-Day Pneumonia/Sepsis-Related Readmission** | **5.7; 95% CI (3.1, 42.0)** |

NS = Non-significant NNT value

On average, 9.9 (95% CI (5.2, 100.4)) patients would have to receive ceftolozane/tazobactam instead of CZA for one additional patient to NOT have **Recurrent MDR-PSA PNA**

On average among hospital survivors, 5.7 (95% CI (3.1, 42.0) patients would have to receive ceftolozane/tazobactam instead of CZA for one additional patient to NOT have a **60-Day Pneumonia/Sepsis-Related Readmission.**

**Supplemental Table 10.** Discharge Destination Between Ceftolozane/Tazobactam and Ceftazidime/Avibactam in the Overall Study Population

|  |  |  |  |  |
| --- | --- | --- | --- | --- |
| **All Patients** | C/T (n) | % | CZA (n) | % |
| Home | 12 | 10.3% | 4 | 5.0% |
| Home w/ Health Service Care | 17 | 14.5% | 2 | 2.5% |
| SNF/ICF | 25 | 21.4% | 36 | 45.0% |
| LTC | 25 | 21.4% | 9 | 11.3% |
| Other Institution (Short Term Care, Rehab Center, Cancer Center, Swing Bed, Other) | 8 | 6.8% | 3 | 3.8% |
| Hospice | 9 | 7.7% | 8 | 10.0% |
| Died | 21 | 17.9% | 18 | 22.5% |
|  |  |  |  |  |
| **Survivors Only** | C/T (n) | % | CZA (n) | % |
| Home | 12 | 12.5% | 4 | 6.5% |
| Home w/ Health Service Care | 17 | 17.7% | 2 | 3.2% |
| SNF/ICF | 25 | 26.0% | 36 | 58.1% |
| LTC | 25 | 26.0% | 9 | 14.5% |
| Other Institution (Short Term Care, Rehab Center, Cancer Center, Swing Bed, Other) | 8 | 8.3% | 3 | 4.8% |
| Hospice | 9 | 9.4% | 8 | 12.9% |

**References**

1. PINC AI™ Healthcare Data White Paper: Data that informs and performs, September 14, 2021. PINC AI™ Applied Sciences, Premier Inc. <https://offers.premierinc.com/rs/381-NBB-525/images/Premier-HealthcareDatabase-Whitepaper-Final.pdf>

2. Deyo RA, Cherkin DC, Ciol MA. Adapting a clinical comorbidity index for use with ICD-9-CM administrative databases. *J Clin Epidemiol* 1992; **45**: 613-9.

3. Kadri SS, Adjemian J, Lai YL et al. Difficult-to-Treat Resistance in Gram-negative Bacteremia at 173 US Hospitals: Retrospective Cohort Analysis of Prevalence, Predictors, and Outcome of Resistance to All First-line Agents. *Clin Infect Dis* 2018; **67**: 1803-14.

4. Kollef MH, Nováček M, Kivistik Ü et al. Ceftolozane–tazobactam versus meropenem for treatment of nosocomial pneumonia (ASPECT-NP): a randomised, controlled, double-blind, phase 3, non-inferiority trial. *The Lancet Infectious Diseases* 2019; **19**: 1299-311.
